# Does sperm DNA fragmentation correlate with semen parameters?

**DOI:** 10.1002/rmb2.12297

**Published:** 2019-09-03

**Authors:** Minh Tam Le, Tam An Thi Nguyen, Hiep Tuyet Thi Nguyen, Thai Thanh Thi Nguyen, Van Trung Nguyen, Dinh Duong Le, Vu Quoc Huy Nguyen, Ngoc Thanh Cao

**Affiliations:** ^1^ Department of OBGYN Hue University of Medicine and Pharmacy Hue University Hue Vietnam; ^2^ Center for Reproductive Endocrinology and Infertility Hue University of Medicine and Pharmacy Hue University Hue Vietnam; ^3^ Department of Histology and Embryology Thai Nguyen University of Medicine and Pharmacy Thai Nguyen Vietnam; ^4^ Department of Public Health Hue University of Medicine and Pharmacy Hue University Hue Vietnam

**Keywords:** chromatin, DNA fragmentation, infertility, semen analysis, spermatozoa

## Abstract

**Purpose:**

This study aimed to investigate the association between sperm quality assessed by routine semen analysis and sperm DNA integrity assay.

**Methods:**

In our cross‐sectional study, a total of 318 men from the infertile couples were enrolled from December 2017 to March 2019 at the Hue Center for Reproductive Endocrinology and Infertility, Vietnam. General characteristics and semen parameters were detected. The sperm DNA fragmentation index (DFI) was estimated by the sperm chromatin dispersion (SCD) assay. A threshold of DFI 30% was applied to classify normal (DFI < 30%) or abnormal (DFI ≥ 30%) groups. The correlations between DFI and semen parameters were analyzed by Spearman's rank correlation coefficient.

**Results:**

In the correlation analysis, DFI was significantly correlated with abnormal head and progressive motility, with a positive correlation with abnormal head (*ρ* = .202, *P* = .0003) and a weak negative correlation with progressive motility (*ρ *= −.168, *P* = .0027), respectively. In the bivariate analysis, DFI was associated with male age, smoking, and alcohol consumption with *P* < .05.

**Conclusions:**

The sperm DFI was not strongly correlated with conventional semen parameters. Therefore, a sperm DNA fragmentation assay should be performed as an additional step in the investigation of male fertility.

## 1 INTRODUCTION

The success of pregnancy is influenced by both men and women. Of all infertility cases, nearly 50% are due to the male factor of infertility, either as a single factor or in combination with the female factor.^1^, ^2^ Male infertility is determined by the quality of the spermatozoa, which affects their ability for fertilization. In infertility cases, a semen analysis that evaluates sperm concentration, motility, and morphology is performed as a standard diagnostic tool to assess sperm quality (WHO, 2010).^3^ In 1991, it was reported that abnormal sperm morphology not only impacted successful fertilization rates and pregnancy rates per cycle but also increased the risk for miscarriages, even if embryo transfer was successful through in vitro fertilization (IVF) cycles.^4^

DNA fragmentation is expressed as the DNA fragmentation index (DFI). DNA fragmentation rates often correlate with semen analysis parameters through a high abnormal DFI (>30%) and may be found in up to 8% of infertile men with a normal semen analysis, suggesting an adjunct role for the standard semen analysis.^5^ In studies of natural pregnancy rates stratified by DFI, the rates of conception were statistically lower among couples with an elevated DFI.

In recent years, a number of tests were introduced for the evaluation of sperm chromatin structure, including TUNEL (terminal dUTPnick‐end labeling), the COMETtest (single cell gel electrophoresis), the AO (acridine orange) test, the CMA3 (chromomycin A3) test, the SCSA (sperm chromatin structure assay) test, and the SCD (sperm chromatin dispersion) test.^6^, ^7^ Except SCD test, most fragment DNA sperm tests require advanced equipments and high cost. The difference between fragmented and nonfragmented sperm was distinguished from conventional microscopes. The SCD test is based on the principle that sperm with fragmented DNA fail to produce the characteristic halo of dispersed DNA loops that is observed in sperm with nonfragmented DNA.^8^ The SCD test is a very simple, rapid, and accurate procedure to determine SDF.^9^ In addition, the SCD test method was equivalent or more sensitive to analysis sperm DNA fragmented than the TUNEL method.^2^ Deproteinized nuclei create the halos of dispersed DNA that correspond to relaxed DNA loops attached to the residual nuclear structure nuclei with fragmented DNA that produce either small halos or no halos of dispersed DNA. In contrast, the sperm nuclei without DNA fragmentation release DNA, creating large halos, or slightly fragmented DNA, creating medium halos.^10^

By routine semen analysis, the normal parameters, including sperm concentration, motility, and morphology, does not ensure normal sperm DNA. However, fertilization can occur even with damaged DNA, resulting in subsequent unsuccessful pregnancy outcomes. The relationship between sperm morphology and the degree of sperm DNA damage has not yet been clearly understood. This study aimed to investigate the association between sperm quality assessed by routine semen analysis and sperm DNA integrity assay.

## 2 MATERIALS AND METHODS

### 2.1 Study design

A cross‐sectional study design was carried out at the Center for Reproductive Endocrinology and Infertility, Hue University of Medicine and Pharmacy, Vietnam from December 2017 to March 2019. Inclusion criteria were men from infertile couples diagnosed with infertility according to the World Health Organization (WHO) standard, with semen analysis and halosperm test results. Exclusion criteria included any cases that were unable to ejaculate, sperm from cryopreservation or surgery, patients with extremely low sperm counts (under 1 million/mL), patients with severe varicocele or azoospermia. Men with general infections or urogenital infections, with retrograde ejaculation, or with history of surgery on testis, of inguinal hernia or of varicocele were also excluded from the study. All the patients agreed to participate in this study and signed the informed consent form. The present study was approved by the Ethics Committee of Hue University of Medicine and Pharmacy.

### 2.2 Clinical approaches

General information was recorded regarding age, occupation, geography, infertility duration, type of infertility, history of internal diseases or surgery, smoking, and alcohol resumption. Physical examination was performed to measure the BMI, waist‐hip circumference, and male genital examination concerning any abnormalities in the penis, scrotum, and testis.

### 2.3 Laboratory procedure

#### 2.3.1 Semen analysis

A semen sample was collected and analyzed following the WHO standard 2010.^3^ Microscopic examination assessed the sperm motility, vitality, concentration, and sperm morphology.

##### Sperm motility

The sperm motility parameter was analyzed by manual counting under phase‐contrast microscopy (Primo Star, Zeiss) at 400x total magnification. The sperm motility is of two categories: progressive motility and nonprogressive motility. In this study, the progressive motility of 200 sperms was assessed.

##### Sperm vitality

The vitality parameter was assessed by the eosin technique under phase‐contrast microscopy (Primo Star, Zeiss) at 400× total magnification as recommended by the WHO. Two hundred cells were counted immediately following liquefaction of the semen samples, and the percentage of viable cells was calculated.

##### Sperm morphology

This parameter was estimated by Giemsa staining. The morphology of sperm head shape and size, acrosomal region, sperm neck, midpiece, tail, and cytoplasmic droplets was determined under microscopy (Zeiss) at 1000× total magnification, according to the 5th edition of the WHO guidelines. At least, 200 sperms were counted to calculate the percentage of both normal and abnormal morphology.

#### 2.3.2 DNA fragmentation test

All semen samples were analyzed for fragmented DNA by Halosperm® HT‐HS10. Halosperm® is based on the sperm chromatin dispersion (SCD) technique, provided by Halotech (Halotech DNA SL), involving a controlled DNA denaturation process to facilitate the subsequent removal of the proteins contained in each spermatozoon. In this way, normal spermatozoa create halos formed by loops of DNA at the head of the sperm, which are not present in those with damaged DNA.

Semen was diluted in culture medium to obtain a maximum concentration of 20 million spermatozoa per milliliter. Aliquots of 0.2 mL of fresh sample semen were diluted in medium to obtain sperm concentrations that ranged between 5 and 10 million/mL. The sperm sample was immersed in agarose microgel and spread onto the slide. After the agarose cooled and polymerized, the lamen was removed, and the slide was dipped in a denaturation solution for 7 minutes. The denaturant solution (AD) was prepared by adding 80 µL of the contents of the acid denaturation solution (Tube labeled AD) to 10 mL of distilled water, mixed and placed in an incubation tray. The sample was incubated in lysis solution for 25 minutes. Subsequently, the slide was washed with distilled water, dehydrated in sequential 70%, 90%, and 100% ethanol baths (2 minutes each), and air‐dried. Finally, the sperm were stained with Giemsa and washed with water. After air‐drying, each slide was examined under a Primo Starlight microscope (Carl Zeiss) at 400× magnification, and 500 sperm were scored.

**Figure 1:**
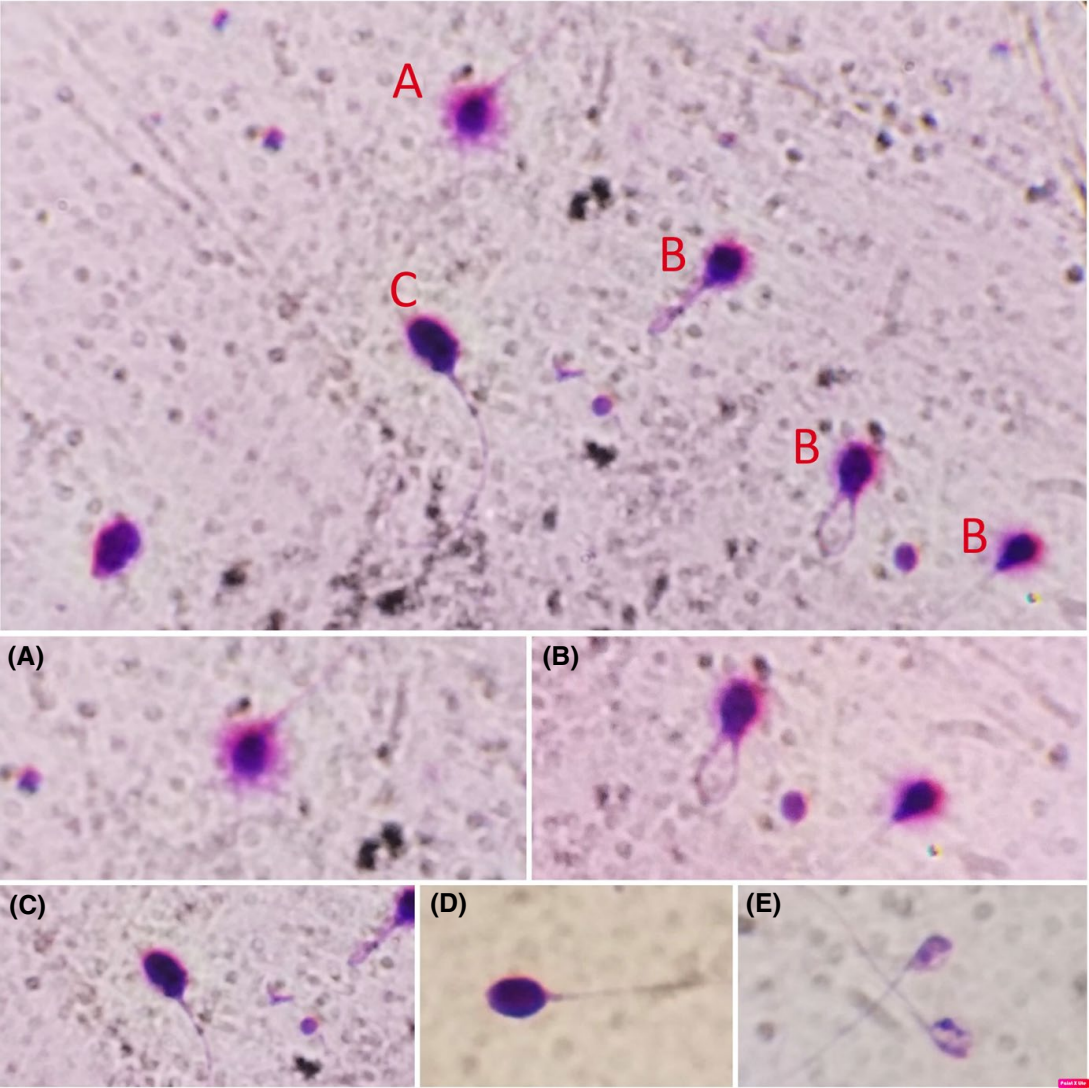
Classification of human sperm DNA fragmentation using Halosperm test. A, Big/large halo: halo width ≥ the diameter of the score; B, Medium halo: small halo ≤ medium halo ≤ big halo; C, small halo: halo width ≤ 1/3 of the diameter of the score; D, Without halo: no halo; E, Degraded: no halo and presence of a score irregularly or weakly stained

The images of halos generated with Halosperm® are highly contrasted and can be evaluated precisely using conventional Primo Star microscopy (Carl Zeiss). Five SCD patterns are possible as shown in Figure 1:

- Sperm cells with large halos (thickness equal to or greater than the length of the minor diameter of the core)
- Sperm cells with medium halos (thickness smaller than the length of the minor diameter of the core and greater than 1/3 of the minor diameter of the core)
- Sperm cells with small halos (thickness equal to or less than 1/3 diameter of the minor diameter of the core)
- Sperm without halos
- Sperm cells with degradation

A total of 500 spermatozoa were counted, and those with DNA fragmentation were identified according to the manufacturer's instructions, and the DNA fragmentation index (DFI) was calculated as indicated below.

$$\text{DFI}\;\left(\%\right)\;=\;100\;\times \;\frac{\text{No}.\;\text{of}\;\text{spermatozoa}\;\text{with}\;\text{fragmented}\;\text{DNA}}{\text{No}.\;\text{of}\;\text{spermatozoa}\;\text{counted}}$$

### 2.4 Statistical analysis

The studied population of men was divided into two groups regarding the DFI value. Based on the recommendation of Halosperm® provided by Halotech (Halotech DNA SL), the DFI threshold was fixed at 30% to distinguish between two groups: DFI ≥ 30% group and DFI < 30% group. This threshold has been used by other authors, who have indicated that DFI levels above 30%, as measured by SCD and sperm chromatin structure assay (SCSA), showed a negative correlation between sperm DNA fragmentation and semen parameters.^11^, ^12^

All statistical analyses were performed using SPSS software (version 22.0, SPSS Inc). All numeric data are presented as the mean value ± standard deviation. Frequencies were expressed as percentages by comparison with mean values among two groups using an analysis of variance test. The association of the standard parameters and the DFI was measured by Pearson's correlation coefficient (*r*). The Wilcoxon rank sum test and Kruskal‐Wallis test were applied to compare the median value for two groups or several groups. Differences between the values were considered statistically significant when *P* < .05.

## 3 RESULTS

A total of 318 men in infertile couples were recruited for the study group. Table 1 shows the general characteristics and DFI results. There are 69 patients with DFI ≥ 30% (21.7%) and 249 patients with DFI < 30% (78.3%) with the maximum DFI is 3.6% and minimum DFI is 99.2%. The DFI was significantly related to male characteristics, such as age, smoking, and alcohol assumption with *P* < .05. The DFI was not found to be significantly different based on the infertility type, infertility duration, body mass index (BMI), occupation, and geography.

**Table 1:** General characteristics of study population and DNA fragmentation index

Characteristics	Total	Mean ± SD	DFI (%) Median (IQR)	*P* value
Age				
≥35 years	155 (48.74)	23.12 ± 18.04	18 (10.6‐29)	.032
<35 years	163 (51.26)	19.97 ± 16.89	14.2 (9‐24)	
Infertility type				
Primary	215 (67.6)	22.0 ± 17.81	16.8 (9.6‐27.6)	.378
Secondary	103 (32.4)	20.49 ± 16.88	13.8 (9.4‐24.6)	
Infertility duration				
>3 years	152 (47.8)	21.32 ± 17.29	15.3 (10‐24.9)	.985
≤3 years	166 (52.2)	21.68 ± 17.75	16.5 (9.6‐28.6)	
BMI kg/m^2^)				
<18.5	9 (2.83)	20.13 ± 12.98	13.4 (9.8‐29.6)	.303
18.5‐22.9	143 (44.97)	20.83 ± 18.74	14.8 (8‐25)	
23‐24.9	71 (22.3)	21.18 ± 16.36	15 (9.4‐30.6)	
≥25	95 (29.87)	22.91 ± 16.91	18.6 (12.4‐29)	
Geography				
Urban	133 (41.82)	22.06 ± 15.99	17.4 (11.8‐27.6)	.166
Rural	185 (58.18)	21.11 ± 18.54	14.4 (9.2‐25)	
Smoking				
Yes	123 (38.68)	23.72 ± 16.16	20.6 (12.4‐31.4)	.001
No	195 (61.63)	20.11 ± 18.20	13.6 (9‐24.4)	
Alcohol assumption				
Yes	142 (44.65)	23.49 ± 18.03	18.6 (11.4‐29)	.013
No	176 (55.35)	19.91 ± 16.94	14.1 (8.2‐25.2)	
DFI				
<30				
≥30				

Abbreviations: BMI, body mass index; mean interquartile range; DFI, DNA fragmentation Index.

Table 2 showed that mean DFI was 14.10% ±6.78% (3.6% ‐ 29.6%) and 48.25% ± 18.26% (30.4% −99.2%) for group DFI < 30% and DFI > 30%, respectively. For each semen parameter tested, there was no significant difference between the obtained values in the two groups regarding pH, volume, concentration, progressive motility, viability, abnormal morphology, and abnormal tail‐neck. Head abnormalities were higher in the DFI > 30 group than in the DFI < 30 group (87.30 ± 5.56 vs 85.22 ± 4.87, respectively).This difference was statistically significant with *P* = .005.

**Table 2:** Fresh semen analysis and sperm DNA fragmentation index (n = 318)

Sperm parameters	Total Mean ± SD	DFI ≥ 30% (n = 69)	DFI < 30% (n = 249)	*P* value
pH	7.09 ± 0.27	7.03 ± 0.27	7.11 ± 0.28	.374
Volume (ml)	1.65 ± 0.89	1.54 ± 0.76	1.69 ± 0.92	.240
Concentration (mil/ml)	33.81 ± 13.58	31.74 ± 13.31	34.38 ± 13.63	.118
Progressive motility (%)	31.68 ± 13.25	29.11 ± 13.64	32.39 ± 13.08	.089
Viability (%)	78.86 ± 9.30	78.64 ± 12.30	78.93 ± 8.30	.432
Abnormal morphology (%)	95.69 ± 2.57	95.90 ± 2.80	95.63 ± 2.50	.156
Abnormal head (%)	85.68 ± 5.09	87.30 ± 5.56	85.22 ± 4.87	.005
Abnormal Tail‐neck (%)	61.34 ± 10.23	62.91 ± 10.84	60.90 ± 10.04	.186

Abbreviation: DFI, DNA fragmentation Index.

The dependence of parameter variables (age, volume, concentration, motility, morphology, and vitality) on the DFI variable is shown in Table 3. There was no correlation between DFI and age, volume, concentration, abnormal morphology, abnormal tail‐neck, and vitality. There was only a statistically positive correlation between DFI and abnormal head (*ρ* = .202, *P* = .0003) and a statistically negative correlation between DFI and progressive motility (*ρ *= −.168, *P* = .0027).

**Table 3:** Correlation between age, the values of standard semen parameters, and sperm DNA fragmentation indexes

Factors	DNA fragmentation indexes	
	Correlation coefficient (*ρ*)	*P* value
Age	.095	.093
Volume	−.022	.691
Concentration	−.080	.152
Progressive motility	−.168	.0027
Vitality	−.085	.132
Abnormal Morphology	−.055	.325
Abnormal Head	.202	.0003
Abnormal Tail‐neck	.027	.636

## 4 DISCUSSION

Sperm DNA damage is the major molecular cause of male infertility, which has a negative effect on reproductive outcomes in couples. Recent clinical practice recommendations suggest the potential role of sperm DNA fragmentation assay in specific clinical scenarios. This would expand the potential of sperm DNA fragmentation assay globally as a prognostic and diagnostic tool in various male infertility scenarios and their treatment management.^13^

Semen analysis by testing conventional parameters is the primary method for assessing men fertility, according to WHO guidelines. It is clear that routine semen analysis can provide a limited prediction of man fertility potential and is not always able to explain the cause of male infertility. In fact, many cases of male infertility are caused by sperm DNA defects, which routine semen quality analyses fail to detect.^14^ The relationship between the sperm DNA fragmentation index (DFI) and semen parameters is still unclear and controversial. While some studies reported a good correlation,^15^, ^16^ other studies could not find any association between DFI and human sperm parameters.^17^, ^18^ Currently, although there are several techniques to do that test, the SCD test (Halosperm test) seems to be a popular and available test. SCD test is not only simple and cost‐effective but also inexpensive equipment requirement.^19^ Furthermore, it is reported that SCD test had significant correlation with other test such as TUNEL, acridine orange, or SCSA.^20^

The rolling of sperm DNA materials is mediated by specific proteins that control DNA condensation and decompression over time. DNA must be compressed to be protected from degeneration and fragmentation before decompression to reveal vertical frames for protein synthesis at embryonic development stages.^21^ Spermatozoa from the infertile men group exhibited a high proportion of DNA damage.^22^ Some reports showed a statistically significant correlation between the sperm DNA fragmentation rate (SDC assay) and the following sperm characteristics: sperm motility, morphology, and concentration.^15^, ^23^

Sivanarayana et al reported that sperm with DNA fragmentation showed a negative correlation with semen parameters: concentration, motility, and normal morphology were significantly lower in the abnormal DNA group (DFI ≥ 30%) than in the normal DNA group (DFI < 30%).^11^ Muriel et al indicated a negative correlation between cells with degraded chromatin and sperm morphology (*r *= −.29, *P* = .04).^24^ Furthermore, the percentage of sperm with progressive motility in semen was negatively correlated with the percentage of cells with a small halo (*r *= −.22, *P* = .04) and positively correlated with the percentage of sperm cells with a large halo (*r* = .30, *P* < .01), indicating a link between progressive motility and intact DNA. Overall, sperm DNA damage was negatively correlated with sperm motility.^24^

In contrast, other studies found no statistically significant correlations between the conventional semen parameters and the degree of sperm DNA fragmentation.^17^, ^18^ Even in men with oligozoospermia, there were no significant correlations between sperm DNA fragmentation and progressive motility, concentration, or morphology.^25^ DFI was correlated with only one of the parameters, such as morphology 26, 27 or motility.^25^ Our results in men from infertility couples showed no correlation between DFI and volume, concentration, or vitality, but DFI and progressive motility showed a negative correlation.

Regarding sperm morphology, head abnormalities, especially amorphous heads, are reported to be related to the elevated degree of DNA fragmentation.^28^ The percentages of normal nuclear sperm showed a significant negative correlation with the percentage of DNA fragmentation.^29^ By evaluating sperm head shape using elliptic Fourier analysis and detecting DNA fragmentation by TUNEL assay, it was concluded that sperm heads with abnormal ellipticity, angularity, and large nuclear vacuoles are associated with DNA fragmentation.^30^

In our study, there was a statistically significant difference in abnormal heads between the DFI ≥ 30% and DFI < 30% group (87.30 ± 5.56 vs 85.22 ± 4.87, *P* = .005, respectively) and a positive correlation between DFI and abnormal sperm head (*r* = .202, *P* = .0003). However, we did not find any relationship between DFI and abnormal morphology or abnormal tail‐neck. In the acrosome phase of spermiogenesis, the head of the developing sperm contains the acrosome and the condensing nucleus, while the growing axoneme extends to become the tail. Flagellum growth continues as the tail and mitochondria aggregate around the proximal region to form a thickened middle piece where the ATP for flagellar movements is generated.^31^ Negative effects at this stage will lead to the abnormal morphology of spermatozoa and DNA damage but may not affect the neck‐tail. Therefore, DFI may be related to sperm head abnormalities but is not related to neck‐tail abnormalities.

Epigenetic changes and DNA mutations along with chromosomal aneuploidies have been associated with increasing paternal age.^32^ The conventional semen analysis can often fail to detect a defect in spermatogenesis (high DFI) in older men and suggest that infertile couples with advanced paternal age, including those with normal semen parameters, should consider sperm DNA testing as part of the couple evaluation.^33^, ^34^ In the present study, we found a significant difference between sperm DNA fragmentation based on age in the two groups (≥35 years vs <35 years). However, in the multivariate analysis, the correlation between DFI and age was not statistically significant (*r* = .095, *P* = .093).

Stress and lifestyle may affect sperm DNA damage.. In clinical studies, the relationship between smoking and sperm DNA fragmentation has been discussed in controversial conclusions.^17^, ^35^ In this study, we found a significant effect of smoking on sperm DNA fragmentation with the DFI of smokers compared with nonsmokers: 23.72 ± 16.16 compared with 20.11 ± 18.20.

Human studies have revealed that alcohol consumption causes significant morphological changes in sperm, leading to abnormal head and tail sperm.^36^ A study by Komiya et al showed that the DFI was significantly different based on alcohol status, and chronic alcohol use increased the DFI by 49.6 ± 23.3% compared with 33.9 ± 18.0% in those who did not regularly consume alcohol (*P* = .0084).^35^ Our results showed that the DFI (23.49 ± 18.03) was increased in the semen samples from those with alcohol use (n = 142) compared with those who did not regularly consume alcohol (19.91 ± 16.94) (*P* = .0132).

In conclusion, our data showed that the sperm DNA fragmentation index was not strongly correlated with conventional semen parameters. Therefore, the sperm DNA fragmentation assay should be performed as an additional step in the investigation of male fertility. Assessment of sperm fragmentation DNA is especially necessary for advanced age patients and men with risk factors such as smoking, alcohol exposure, or sperm with high rate of abnormal head.

## DISCLOSURES

*Conflict of interest*: The authors report no conflicts of interest. The authors alone are responsible for the content and writing of this article. *Human rights statements and informed consent*: Informed and written consent was obtained from all participants. This study was approved by the Hue University of Medicine and Pharmacy Ethics Committee, Hue University, Vietnam.
